# Supplementary material for: Hydroxyl super rotors from vacuum ultraviolet photodissociation of water
Source: Nat Commun. 2019 Mar 19;10:1250. doi: 10.1038/s41467-019-09176-z (PMC6424997; doi:10.1038/s41467-019-09176-z)
Supplement: Supplementary file 1 — Supplementary Information [file 41467_2019_9176_MOESM1_ESM.pdf]

Supplementary Information

Hydroxyl Super Rotors from Vacuum Ultraviolet Photodissociation of Water

Chang et al

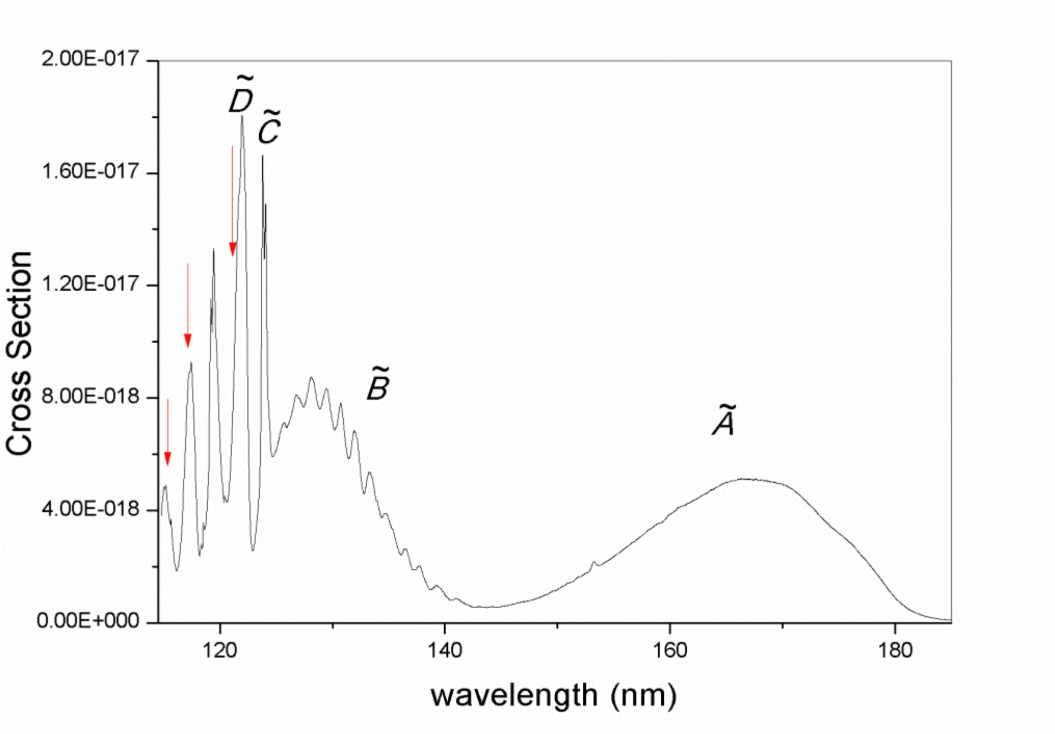

Supplementary Fig. 1 Absorption spectrum of a room temperature sample of H_2O vapor (adapted with permission from ref.1. Copyright 2001 American Chemical Society). The photolysis excitation wavelengths used in the present work are indicated by the vertical red arrows.

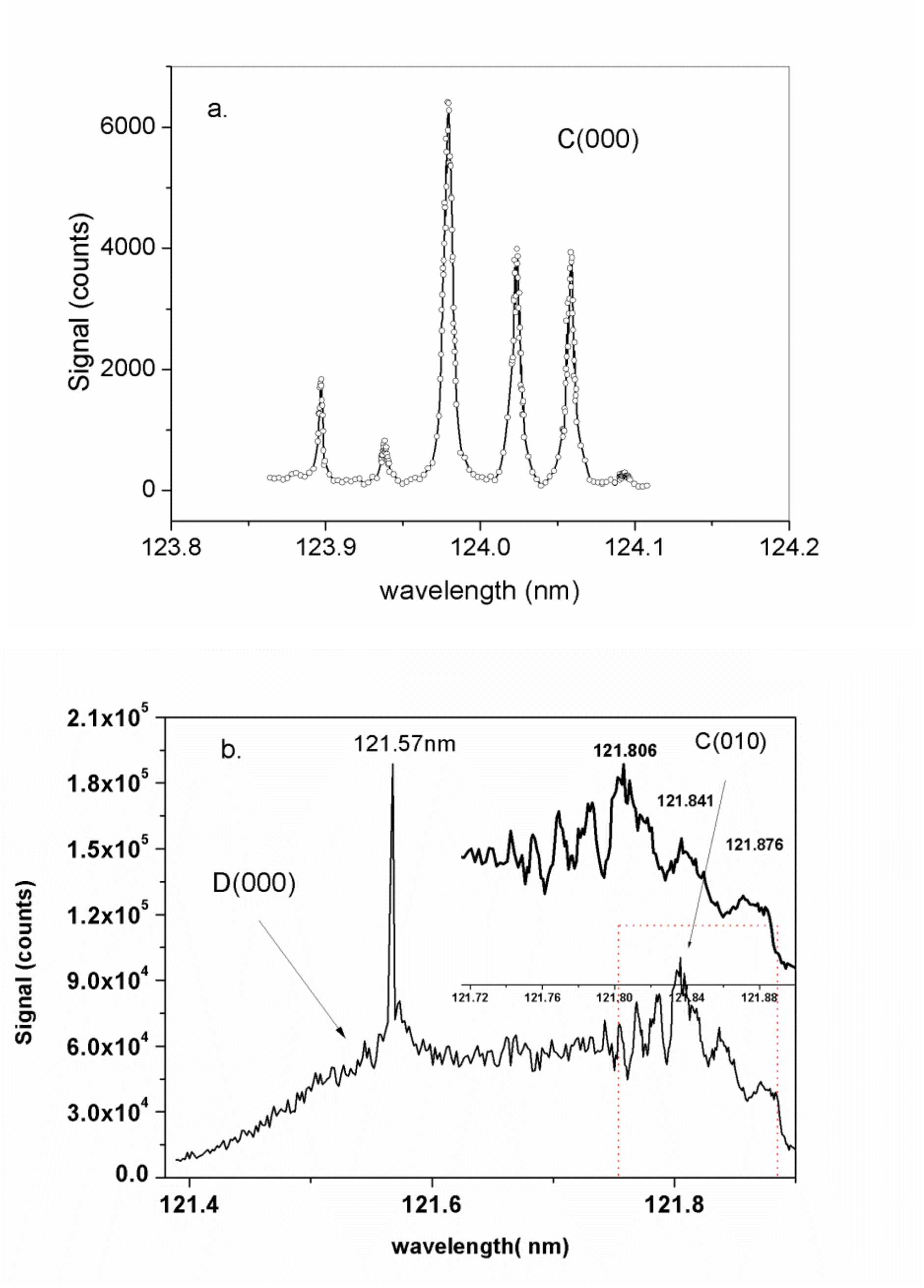

Supplementary Fig. 2 Wavelength resolved action spectra for forming H atoms following photoexcitation of H_2O via (a) the $\tilde{C}$(000) state and (b) the overlapping $\tilde{D}$(000) and $\tilde{C}$(010) states.

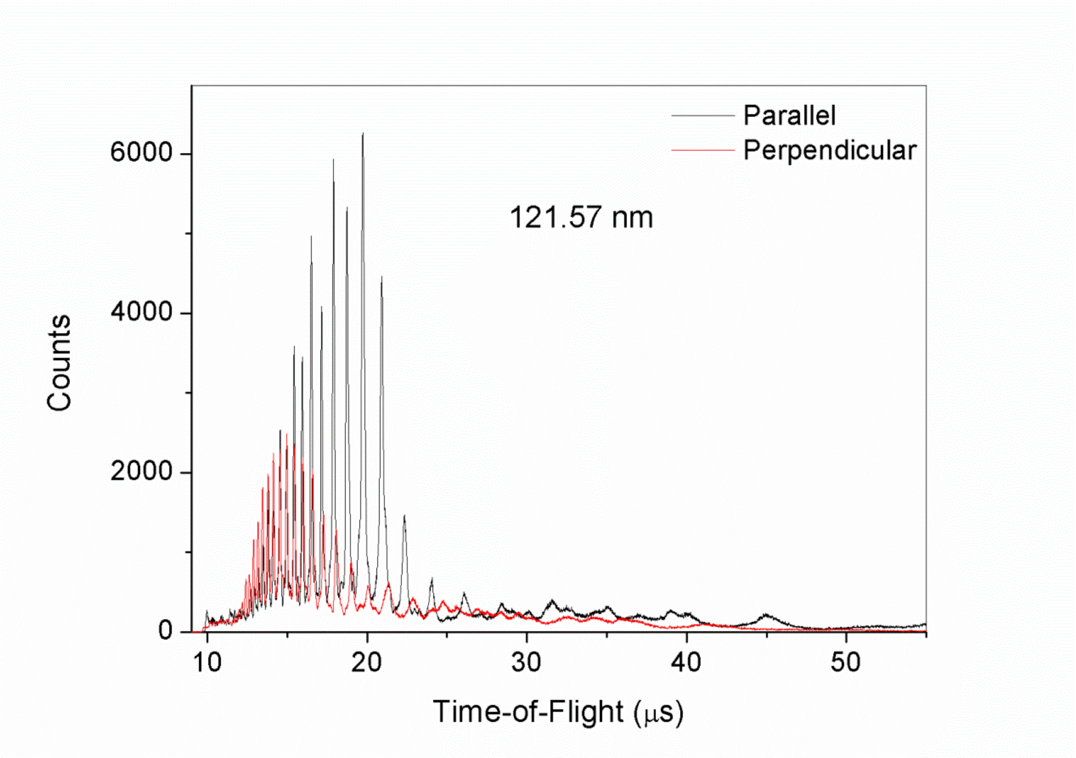

Supplementary Fig. 3 Time-of-flight spectra of the H atom products from photodissociation of H_2O at 121.57 nm (VUV-FEL) with the detection axis parallel (black) and perpendicular (red) to the photolysis laser polarization.

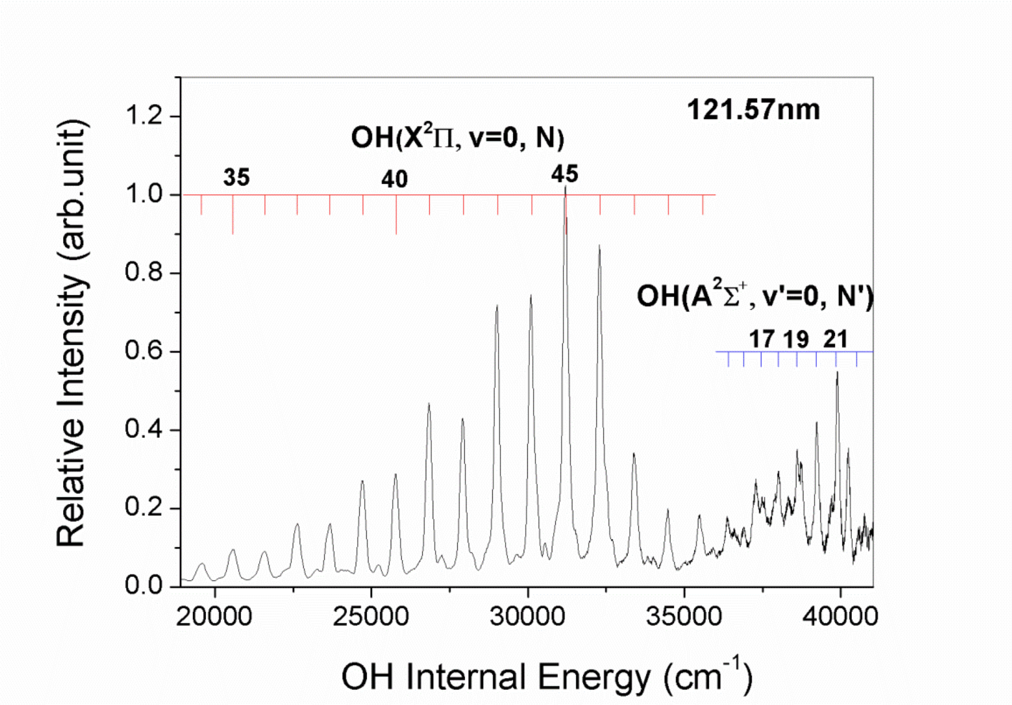

Supplementary Fig. 4 Internal energy spectrum of the OH products from photodissociation of H_2O at 121.57 nm, obtained from H atom TOF spectra recorded with the detection axis parallel to the photolysis laser polarization. The sharp features can all be assigned to population of rovibrational states of OH(*X*) and OH(*A*). The spectral resolution of OH(*A*) is lower than that observed in ref. 2, due to the broad bandwidth of VUV-FEL laser beam (~50 cm^{-1}).

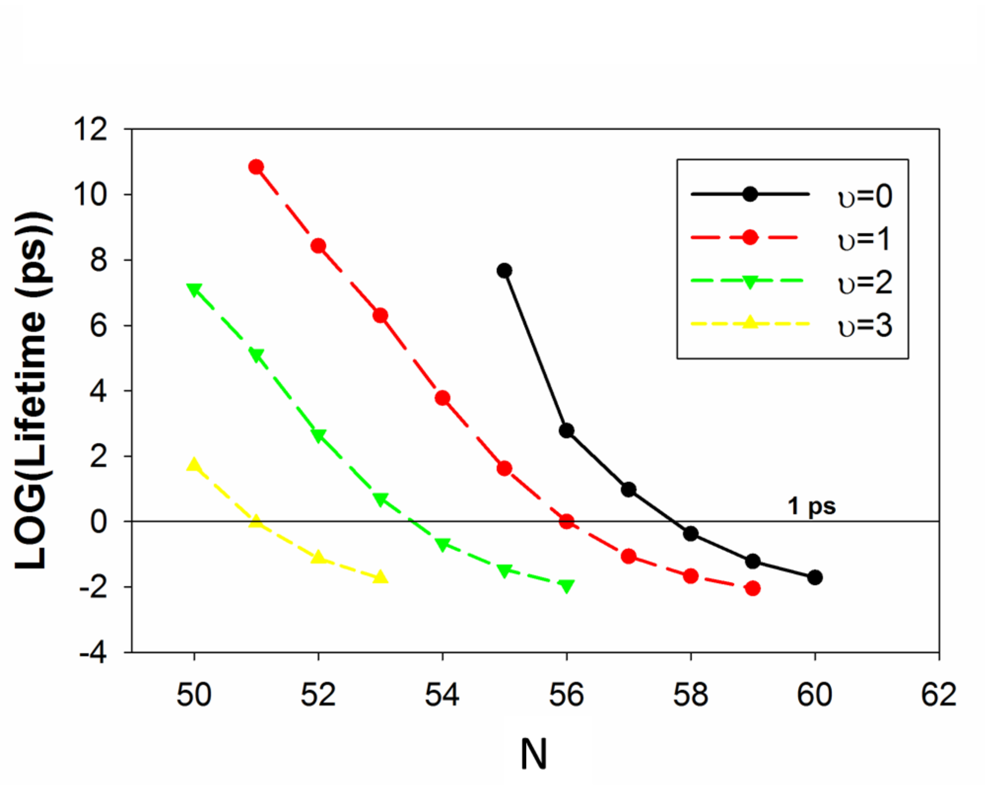

Supplementary Fig. 5 Calculated tunneling lifetimes of super rotationally excited levels of OH(X, v=0, 1, 2, 3) lying above D_0(O–H).

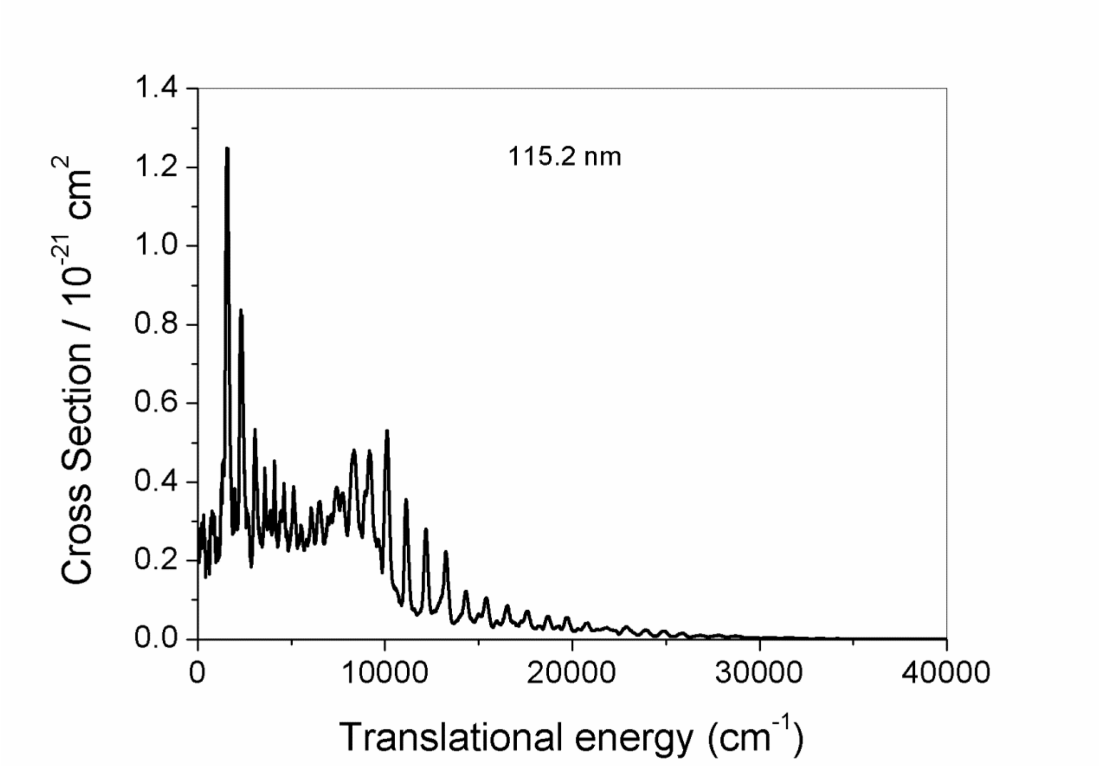

Supplementary Fig. 6 Translational energy resolved differential cross sections for the H atom products formed in the 115.2 nm photodissociation of H_2O with the detection angle of 55° (magic angle). The integrated cross section is assumed to be ~4.4×10^{-18} cm^2.

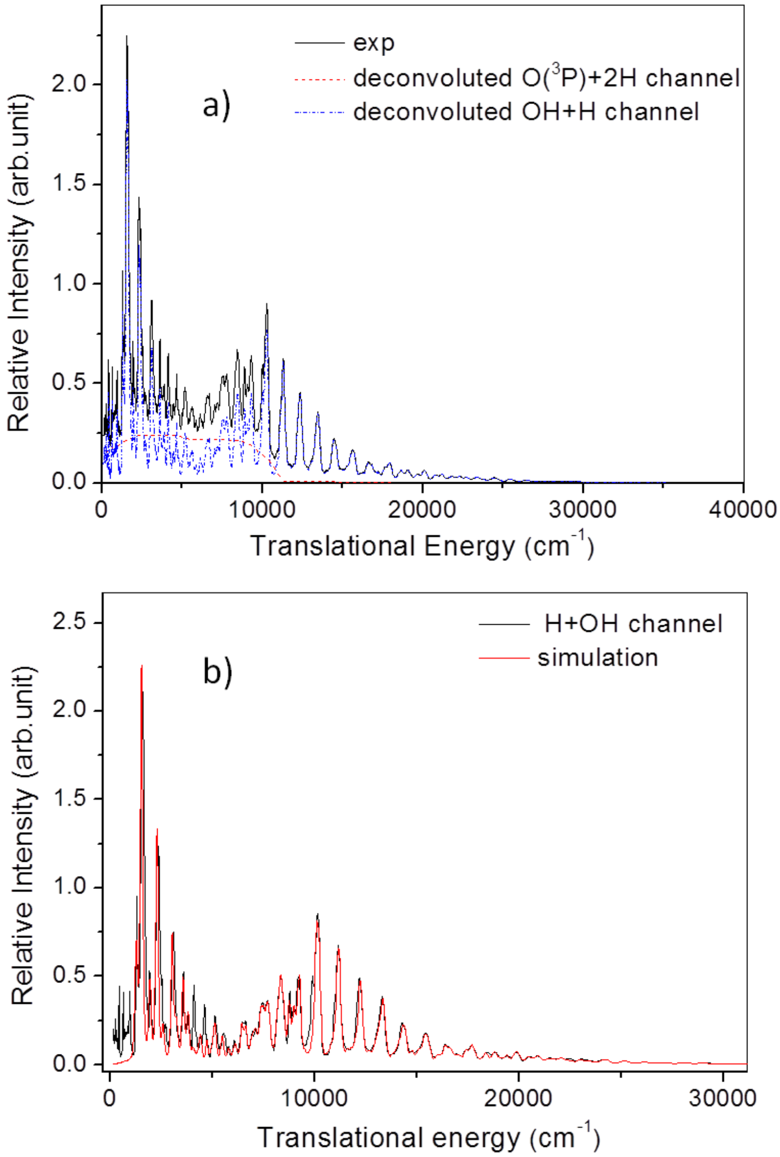

Supplementary Fig. 7 a) Deconvolution of the translational energy spectrum derived from the H atom TOF spectrum measured following 115.2 nm photolysis of H_2O into components associated with H+OH and $O(^3P)$+2H products. b) The deconvoluted H+OH translational energy spectrum and simulated spectrum from 115.2 nm photolysis of H_2O.

Supplementary Note1

The absorption spectrum of H_2O in the wavelength range 190~120 nm consists of two broad, well-separated bands with maxima at ~167 nm and ~128 nm (**Supplementary Fig. 1**). The 190~150 nm and 140~120 nm bands are assigned to, respectively, the $\tilde{A}^1B_1 \leftarrow \tilde{X}^1A_1$ and $\tilde{B}^1A_1 \leftarrow \tilde{X}^1A_1$ electronic transitions; the latter shows a progression of diffuse resonance structures with separations of ~800 cm^{-1}.[3]

Sharper absorption features appear at shorter wavelengths, including the $\tilde{C}^1B_1 \leftarrow \tilde{X}^1A_1$ and $\tilde{D}^1A_1 \leftarrow \tilde{X}^1A_1$ Rydberg transitions with electronic origins at, respectively, 124 and 122 nm.[4] Both show short ($v_1v_2v_3$) vibrational progressions extending below 120 nm (where v_1, v_2 and v_3 represent the number of quanta in the symmetric stretch, bend and antisymmetric stretch modes, respectively). Bell *et al.*[5] originally suggested two possible assignments for the peaks at ~122, 117 and 115 nm, each based on $\tilde{C} \leftarrow \tilde{X}$ and $\tilde{D} \leftarrow \tilde{X}$ transitions to excited state levels with different combinations of v_1, v_2 and v_3. But the $\tilde{C}$ state is adiabatically bound and only relatively weakly predissociated, so absorptions to this state show reasonably sharp structure. Thus, for example, the 'action spectrum' for forming H atoms when exciting with tunable VUV radiation from a four-wave mixing cell locates the $\tilde{C} \leftarrow \tilde{X}$ origin band in the wavelength range 123.9-124.1 nm and the $\tilde{C}(010) \leftarrow \tilde{X}$ transition in the region 121.7-121.9 nm (**Supplementary Fig. 2**). Given the fundamental wavenumbers ν_1=3179 cm^{-1}, ν_2=1407 cm^{-1} and ν_3=3238 cm^{-1} for the $H_2O(\tilde{C})$ state,[6] allows us to predict that the $\tilde{C}(110) \leftarrow \tilde{X}$ and $\tilde{C}(200) \leftarrow \tilde{X}$ transitions should be centred at ~117.26 and ~114.95 nm, respectively. In the present work, the VUV-FEL was tuned to 121.57, 117.5 and 115.2 nm, *i.e.* off-resonant from that required to access these vibrationally excited

levels of the $\tilde{C}$ state. The $\tilde{D}$ state levels, in contrast, are very short lived as a result of efficient non-adiabatic coupling with the $\tilde{B}$ state at bent geometries and the $\tilde{D}$ $(v_1v_2v_3)\leftarrow\tilde{X}$ transitions show no rotational structure. Yuan *et al.*[6] reported a band width of ~390 cm^{-1} for the $H_2O(\tilde{D}\leftarrow\tilde{X})$ origin transition. Thus, the 121.57, 117.5 and 115.2 nm excitation wavelengths used in the present study are confirmed as populating the $\tilde{D}(000)$, $\tilde{D}(100)$ and $\tilde{D}(110)$ states of H_2O, respectively.

The H atom action spectra around 124 nm and 121.8 nm were recorded by using our previously reported experimental method,[7] in which two VUV laser beams were generated using four-wave mixing scheme in the single Kr/Ar gas cell. one VUV beam is fixed at the 121.57 nm wavelength to probe the H-atom product through the high-*n* Rydberg tagging technique, the other beam is tunable for photodissociating molecules in the wavelength range λ_{VUV}=123.8-124.1 nm or 121.4-122 nm. The λ_{VUV} radiation was produced by focusing the tunable laser beam (λ_T=700-870 nm) into the same mixing cell as used for generating 121.57 nm. The relative intensity of the 121.57 nm and the tunable VUV light at λ_{VUV} could be controlled by tuning the relative power of the 845 nm laser and the tunable laser at λ_T. Since the 121.57 nm radiation itself will generate photodissociation signals, the intensity of the tunable VUV light is set to be ~10-times higher than that of 121.57 nm light so that the 121.57 nm background signal can be easily subtracted. When the wavelength of λ_{VUV} was tuned to ~121.57 nm (resonant with Lyman α transition), the λ_{VUV} beam can act as both the photolysis and probe laser beams, leading to a sharp rise in signal ~121.57 nm (**Supplementary Fig. 2**).

Supplementary note 2

Supplementary Fig. 3 shows H atom TOF spectra obtained by photolysis of water at 121.57 nm with the detection axis parallel and perpendicular to the polarization vector of VUV FEL radiation. **Supplementary Fig. 4** shows the TKER spectrum derived from the parallel polarization data in Supplementary Fig. 3. It is clear that all of the intense sharp features in Supplementary Fig. 4 can be readily assigned to high rotational levels of OH (X, v=0) and OH (A, v=0) radicals – similar to that obtained in the present work at λ = 117.5 nm, and to that reported previously by Harich *et al.*[2]

Supplementary note 3

The super rotors, with an energy above the dissociation limit, are stable only through the support of centrifugal barriers. Molecules in these super-rotationally excited levels can dissociate by tunneling through barriers, whereas the tunneling probability is expected to be small. In the WKB approximation[8] this tunneling probability is roughly proportional to the exponential of the area of the barrier above the tunneling energy. Based on the previously reported potential energy curve for OH($X^2\Pi$)[9], the wave functions for super rotationally excited levels of OH($X^2\Pi$, $v \geq 0$), and thus the tunneling lifetimes through the centrifugal barriers, can be calculated from first principles, as shown in **Supplementary Fig. 5**.

Supplementary note 4

Cross sections for forming OH "super rotors" in the 115.2 nm photodissociation of H_2O

The initial excitation of H_2O at 115.2 nm is to the $\tilde{D}$(110) state. As noted in the main paper, efficient non-adiabatic coupling between the $\tilde{D}$ and $\tilde{B}$ state potential energy surfaces (PESs) at bent geometries ensures rapid non-radiative transfer to the $\tilde{B}$ state PES – the topography of which then controls the fragmentation dynamics.

The total photoabsorption cross-section of H_2O at 115.2 nm is $\sigma_{tot} \sim 5\times10^{-18}$ cm^2.[10] Possible dissociation channels for H_2O following excitation at 115.2 nm are listed below, along with the corresponding thermochemical threshold energy;[2]

$H_2O + h\nu \rightarrow H + O(^3P) + H$ 9.494 eV

$H + OH(A^2\Sigma^+)$ 9.124 eV

$O(^1D) + H_2(X^1\Sigma_g^+)$ 6.983 eV

$H + OH(X^2\Pi)$ 5.102 eV

$O(^3P) + H_2(X^1\Sigma_g^+)$ 5.106 eV

The $O(^3P) + H_2(X^1\Sigma_g^+)$ channel is spin-forbidden and not observed experimentally. The spin-allowed $O(^1D) + H_2(X^1\Sigma_g^+)$ channel has not been observed experimentally yet, but is considered to play an important role in cometary atmospheres. van Harrevelt and van Hemert [11] have calculated energy dependent partial cross sections for forming $O(^1D) + H_2$ products and estimated a value of ~0.6×10^{-18} cm^2 at 115.2 nm (10.76 eV). Accepting these values, the total cross-section for forming H+OH(*X*)/OH(*A*) and $O(^3P)$+2H products would be ~4.4

$\times 10^{-18}$ cm^2.

The present TOF measurements of H atoms from the H+OH(X)/OH(A) and O (^{3}P)+2H channels in the photodissociation process are achieved using a sequential two-color VUV-UV (ultraviolet) excitation scheme, which provides near unit H atom detection efficiency. The translational energy resolved differential cross sections for forming H atoms (normalized so that σ_{tot} ~4.4$\times 10^{-18}$ cm^2) are plotted in **Supplementary Fig. 6**. The available spectroscopic data [2,12] for the OH radical allow most of the sharp features in Supplementary Fig. 6 to be assigned to highly rotationally excited levels of OH(X, v) and OH(A, v′). The spectral simulation has been performed using software developed in-house[2], in which a Gaussian profile is used to simulate the population of each quantum state. The width of the profiles used in the simulation varies according to the relative kinetic energy resolution ($\delta E/E$ <1%) and the VUV-FEL beam width. (For example, a width of 150 cm^{-1} has been used in the simulation at TKER ~10000 cm^{-1}). The intensities of each OH quantum state and of the broad feature at low translational energy (attributable to the triple fragmentation to O (^{3}P) + 2 H products) are then adjusted to produce a summed simulated TKER spectrum that best matches the experimental data. This process is complicated by the overlap of rotational levels associated with different vibrational states of the OH product, which can obscure the relative contributions of the different levels in the simulation, but is helped by the expectation that the rotational population distributions associated with each vibrational state should vary relatively smoothly with N.

As **Supplementary Fig. 7** shows, the agreement between the experimental and simulated distributions is good and allows estimation of the fractions of the total H atom yield for the H+OH(X) : H+OH(A) : O(^{3}P)+2H product channels of 0.39 : 0.26: 0.35. Recognizing that two H atoms are formed in the triple dissociation process, the branching ratios for the three channels are 0.39:0.26:0.18. We note that the branching fraction into the triple dissociation channel is least well determined, as this process has not been fully characterized in the experiment (The fitting error bar for triple dissociation channel is estimated to be $\pm$30%). Thus the cross section for H+OH(X) product formation following 115.2 nm photodissociation of H_2O can be estimated to be ~0.47(±0.04)$\times$4.4$\times$$10^{-18}$ cm^2 = 2.1($\pm$0.18)$\times$$10^{-18}$ cm^2. About 30% of the OH(X) products formed at this wavelength are in rotational levels with energies greater than D_0(O–H), suggesting that the cross section for forming OH “super rotors” in the 115.2 nm photolysis of H_2O at is ~(6.3$\pm$0.6)$\times$$10^{-19}$ cm^2.

Supplementary References

1 Fillion, J. H., van Harrevelt, R., Ruiz, J., Castillejo, M., Zanganeh, A. H., Lemarie, J. L., van Hemert, M. C. & Rostas, F. Photodissociation of H_2O and D_2O in B, C, and D states (134-119 nm). Comparison between experiment and ab initio calculations. *J. Phys. Chem. A* **105**, 11414-11424 (2001).

2. Harich, S. A., Hwang, D. W. H., Yang, X. F., Lin, J. J., Yang, X. M. & Dixon, R. N. Photodissociation of H_2O at 121.6 nm: A state-to-state dynamical picture. *J. Chem. Phys.* **113**, 10073-10090 (2000).

3. Weide, K., Kuhl, K. & Schinke, R. Unstable periodic-orbits, recurrences, and diffuse vibrational-structures in the photodissociation of water near 128 nm *J. Chem. Phys.* **91**, 3999 (1989).

4. Wang, H. T., Felps, W. S. & McGlynn, S. P. Molecular Rydberg states .7: Water. *J Chem. Phys.* **67**, 2614-2628 (1977).

5. Bell, S. The spectra of H_2O and D_2O in the vacuum ultraviolet. *J. Mol. Spectrosc.* **16**, 205-213 (1965).

6. Yuan, K. J., Cheng, L. N., Cheng, Y., Guo, Q., Dai, D. X. & Yang, X. M. Two-photon photodissociation dynamics of H_2O via the D electronic state. *J. Chem. Phys.* **131**, 074301 (2009).

7. Yuan, K. J., Cheng, L. N., Cheng, Y., Guo, Q., Dai, D. X. & Yang, X. M. Tunable VUV photochemistry using Rydberg H-atom time-of-flight spectroscopy. *Rev. Sci. Instrum.* **79**, 124101 (2008).

8. Razavy, M. Quantum theory of tunneling. World Scientific Press, 2003.

9. Huxley, P. & Murrell, J. N. Ground-state diatomic potentials. *J. Chem. Soc., Faraday Trans. 2* **79**, 323-328 (1983).

10. Lee, L. C. & Suto, M. Quantitative photoabsorption and fluorescence study of H_2O and D_2O at 50-190 nm. *Chem. Phys.* **110**, 161-169 (1986).

11. van Harrevelt, R. & van Hemert, M.C. Quantum mechanical calculations for the $H_2O+h\nu \rightarrow O(^1D)+H_2$ photodissociation process. *J. Phys. Chem. A* **112**, 3002-3009 (2008).

12. Coxon, J. A. Optimum molecular-constants and term values for the $X^2\Pi$ and $A^2\Sigma^+$ states of OH. *Can. J. Phys.* **58**, 933-949 (1980).
